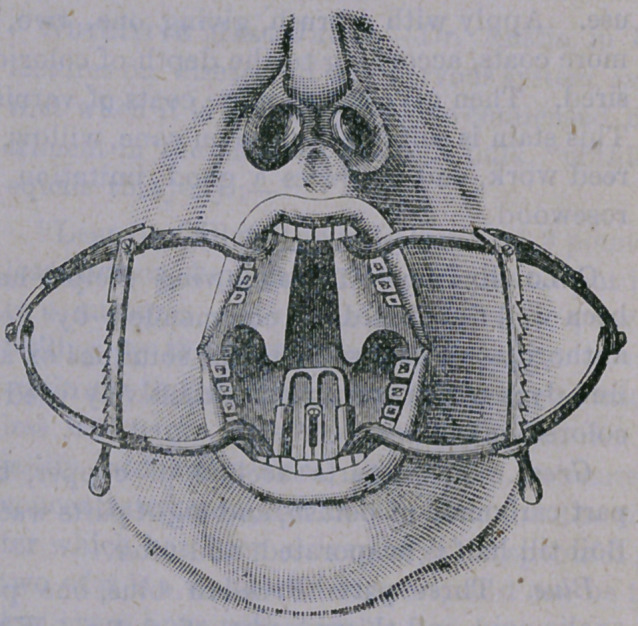# Cleft Palate

**Published:** 1875-01

**Authors:** 


### CLEFT PALATE.

Cleft palate consists, as its name sufficiently indicates, of an opening in the back portion of the roof of the mouth. It is generally accompanied by hare-lip, when the fissure or opening extends through the lip and upper jaw, clear through to the back of the mouth, causing an unsightly and disagreeable deformity. Children are born with this difficulty, and when accompanied with hare-lip, should be early operated upon, with a view to removing the horrible deformity of the features. Cleft palate, alone, causes but little inconvenience, until the child attempts to speak, when its articulation will be so imperfect as to render it very difficult to be understood. The fissure can be closed at any period of life. We have operated upon children of six years of age, as well as adults of sixty-five. Any age is therefore suited to the cure of cleft palate. In order to give our readers an oppor tunity for learning how ingenious are the surgeon's devices for accomplishing such difficult surgical operations as that of sewing up a cleft in the roof of one's mouth, we have only here to introduce a very fine illustration of a "mouth gag," used in this operation, for the purpose of holding the mouth open, and at the same time depressing the tongue, while the surgeon is at work. At the back and top of the mouth will be seen the large opening, and the parts in position for the operation. The surgeon carefully and quickly pares the edges of the palate, so as to render its surface fresh, and then deftly introduces three or four delicate silver threads, by means of small, bent needles, held in long handles. The threads being all introduced, they are brought together, and the edges of the palate approximated, when the opening becomes completely closed. The threads are then twisted and cut close to the knot, and the patient fed upon cold gruel, &c., for four or five days, at the expiration of which time, the wound will have healed and all trace of a cleft palate removed. In this way, the voice is restored, and the patient enabled to speak plainly, and is no more subjected to the mortification of "talking through the nose."

## Figures and Tables

**Figure f1:**